# Retreatment of XP-endo Shaper and R-Endo files in curved root canals

**DOI:** 10.1186/s12903-023-02735-3

**Published:** 2023-01-24

**Authors:** Hayam Y. Hassan, Fahd M. Hadhoud, Ayman Mandorah

**Affiliations:** 1 Endodontic Department, Faculty of Dentistry, Suez Canal University, Ismailia, Egypt; 2 Endodontic Department, Faculty of Dental Medicine Assiut Branch, Al-Azhar University, Assiut, Egypt; 3 Restorative and Dental Materials Department, Faculty of Dentistry, Taif University, P.O Box 11099, Taif, 21944 Saudi Arabia

**Keywords:** Root canal retreatment, Curved root canals, XP-endo Shaper, R-Endo

## Abstract

**Objectives:**

To compare the retreatment efficiency of XP-endo Shaper and R-Endo files in curved root canals using ImageJ software.

**Methods:**

Forty extracted mandibular first molars with curved mesial canals (25°–35°) were chosen. Access cavities, preparation and obturation of root canals were performed. Roots were randomly distributed into two groups corresponding to the retreatment files used. Group 1 retreated with XP-endo Shaper file, and group 2 retreated with R-Endo files. Each root was sectioned and photographed. ImageJ software was used to estimate the percentage of residual filling material that existed on the three root canal segments. Mann–Whitney U and the Kruskal–Wallis tests were used to compare the percentages of residual filling material between the teeth segments (P < .05).

**Results:**

The R-Endo group showed a significantly higher median percentage of residual filling material than the XP-endo Shaper group. In both groups, the coronal segments had the highest median of the residual filling material, followed by the middle and apical segments.

**Conclusion:**

XP-endo Shaper is significantly more effective than R-Endo in removing root canal filling materials in the coronal, middle, and apical segments.

## Introduction

To describe endodontic failure, although the root canal treatment was adequate and there were no caries, marginal staining, and/or leakage of the coronal restorations, the root canal treatments were considered clinically unsuccessful if the patient has complaints from related tooth. In some cases, the related teeth may exhibit persistent/unhealed periapical problem radiographically [1].

Nonsurgical retreatment is intended to improve healing of periapical tissues. Because necrotic tissue and bacteria found in the residual gutta-percha and sealer may cause posttreatment disease, the root canal system must be reopened by eliminating the obturating material and then three-dimensional shaping, cleaning, disinfection, and obturation [2, 3] .

Hand files, heated instruments, Gates Glidden burs, and nickel-titanium (NiTi) rotary files have all been described as tools and techniques for eliminating filling materials [4]. Both rotary instruments and hand files leave residual filling material when used to remove infected obturating materials from severely curved canals and canals with complex anatomies [5].

Several instruments, including changes in cross-sectional design and novel NiTi alloys, have been suggested. The XP-endo Shaper (30/0.04) (La Chaux-de-Fonds, Switzerland) is a snake-shaped instrument created from a martensite MaxWire alloy with a 0.01 taper. When subjected to body temperature, however, it expands to a tip size of 30 and a taper of.04 hanging on the canal anatomy. The XP-endo Shaper (XPS) provides more effective mechanical debridement than conventional shaping systems, resulting in fewer untouched areas [6, 7].

Classical rotary nickel-titanium (NiTi) retreatment systems, such as the R-Endo system (Micro-Mega, Besancon, France), have been shown to improve filling material removal when compared to conventional systems [8].

The objective of this study was to evaluate the retreatment efficacy of XP-endo Shaper and R-Endo in curved root canals by measuring the residual filling material in the three root canal segments using ImageJ software.

## Methods

### Sample selection

The sample size determination was performed for compare retreatment efficiency using G-Power v3.1.3 software (University of Dusseldorf; Dusseldorf, Germany). A power analysis revealed that a sample size of 40 specimens per subgroup was found to meet the constraints of α = 0.05 and power = 0.95.

Forty unidentified first mandibular molars extracted for periodontal reasons were selected from a poole containing extracted teeth. The teeth were extracted at the oral surgery and maxillofacial department in the Faculty of Dentistry, Suez Canal University. All teeth were scanned using cone beam computed topography (CBCT) to include molars that had completely formed apices and mesial roots, with two separate orifices ending in two separate foramina (type IV Vertucci canal configuration). The mesial canal curvature ranged between 25° and 35° according to Schneider’s technique [9]. Teeth with calcified root canals, external or internal root resorption, previous endodontic manipulation, or cracks were excluded.

This study was double-blinded by the operator and the observers. The allocator divided the collected teeth into two groups with coded numbers, and each group was retained in an opaque envelope. The operator was aware of the file type at the time of canal retreatment for the coded grouped teeth. A random sequence was created using computer software (http://www.random.org/) [10].

### Sample preparation

Endo-access burs (Dentsply Maillefer) were used to prepare the access cavities using a high-speed handpiece. Diamond disks (Kerr Dental, Orange, CA, USA) were used for hemi-sectioning of the teeth at the furcation area to remove their distal roots, as the experimental procedures were performed on the mesial roots. A #10K-file (MicroMega, Besancon, France) was inserted into the mesiobuccal and mesiolingual root canals to check for patency until the tip of the instrument was evident at the apical foramen [11].

The chosen samples were serially numbered from one to forty. The preparation procedures were carried out under the magnification of a dental operating microscope (Leica Microsystems, Wetzlar, Germany). To maintain the normal trajectory of the irrigation tools and to afford a reservoir for the irrigant, the crowns of the selected samples were not removed [12]. The crown lengths of the teeth were modified to a standardized length of 19 mm. #10K-files were inserted into the mesial root canals and progressed through the root canal until they became flushed with the apical foramen, and the reference points were adapted at the mesiobuccal and mesiolingual cusps of the tooth.

The working length (WL) was estimated by withdrawing 0.5 mm for each root canal, and all measurements were recorded with a triangular architect's scale ruler [13].

All canals were serially prepared to the working length using ProTaper Next (PTN) (Maillefer, Dentsply, Ballaigues, Switzerland), X1(17/0.04), and X2 (25/0.06). ProTaper Universal SX (Dentsply Maillefer) was initially used to prepare the coronal part. The files were operated using an endodontic motor (X-Smart; Dentsply Maillefer) at 300 rpm and 4–5.2 Ncm with a 16:1 reduction handpiece [14].

After each successive file size, 2 mL of 2.5% sodium hypochlorite (NaOCl) irrigation was used. The irrigant was delivered using a plastic syringe with a blunt-end-side vented 30-gauge needle introduced 2 mm short of root length. All roots were irrigated with 1 mL 17% ethylenediaminetetraacetic acid (EDTA) (META, BIOMED CO. LTD, Korea.) for at least 1 min as the final rinse [15]. Finally, all root canals were irrigated with 2 mL of sterile distilled water to remove any debris from the canals.

Obturation of all root canals was performed with gutta-percha points and Sure-Seal RootTM (Sure-Endo, Korea) bioceramic-based root canal sealer supplied in a 2 mL automix syringe. The selected X2 (25/06) (Dentsply, Tulsa, Tulsa, Okla) master cone was covered with sealer and placed on the WL. Fine-medium accessory cones (Dentsply Maillefer) were laterally compacted to complete obturation procedures using standardized # 25 finger spreaders (Dentsply Maillefer, Ballaigues, Switzerland). Gutta-percha excess was cut from canal orifices using a heated instrument [16]. All roots were radiographed in the buccolingual and mesiodistal direction using digital radiography system to confirm the obturation quality. The roots were then incubated at 37 °C and 100% humidity for at least seven days to perform complete setting.

### Retreatment procedures

The samples were then randomly allocated into two equal groups of 20 roots, each containing 40 mesial root canals, rendering the type of files used in root canal filling material removal. The XP-endo Shaper file was used for retreatment of all the mesiobuccal canals, while the R-Endo system was used for retreatment of all the mesiolingual canals.

#### XP-endo Shaper file (XPS)

The roots were mounted on 20 glass vials and immersed in a warm water bath at 37 °C to mimic physiological conditions. The filling materials were removed by creating a start in the coronal portion of the canal orifice (3–5 mm) using a D-Race (DR1) (30/0.10) (FKG Dentaire SA, La Chaux-deFonds, Switzerland) operating at 800–1000 rpm and 1.5 Ncm. The tip of XPS (30) was introduced into the prepared space in the gutta-percha, slightly disengaged, and the motor (Tri Auto ZX2, J. MORITA, Japan) was set at 3000 rpm and 1 N cm. This results in a greater scrubbing of the canal walls. A pecking motion was used until the tip of the XPS file engaged the gutta-percha, and light pressure was then applied to advance the file down the canal to the full WL. An additional 10–15 long strokes to the full WL were used to allow the file to gently corkscrew around the gutta-percha and remove it coronally [17].

#### R-Endo retreatment files

The filling materials were removed using the retreatment R-Endo files. The R1 file (25/0.08) was used in the coronal segment, whereas the R2 file (25/0.06) was introduced until the beginning of the middle segment of the canal. The R3 file (25/0.04) was then operated until full WL was reached. The speed used was fixed at 350 rpm and 1.5 N/cm [18].

### Final irrigation protocol

The removal process was considered complete if there were no noticeable obturating materials on the file after instrumentation procedures. It is imperative to note that during removal of the obturating material, no solvents were used, and the canals were irrigated with 2 mL of 2.5% NaOCl using 30-gauge syringes up to 2 mm short of the WL after each file followed by 1 mL EDTA then finally using 2 mL of sterile distilled water. All files were used as directed by the manufacturer and were discarded after use in the two canals.

### Evaluation of residual filling material using stereomicroscope

With a pointed, narrow, high-speed tungsten carbide bur and copious water cooling, two opposing longitudinal grooves were prepared along the external mesial and distal root surfaces to simplify the subsequent splitting of the root to show the instrumented canal. The grooves were carefully prepared to avoid iatrogenic canal space perforation, which would introduce a burst of contaminants from the water spray and dentin chips into the canal space [19].

**Fig. 1:**
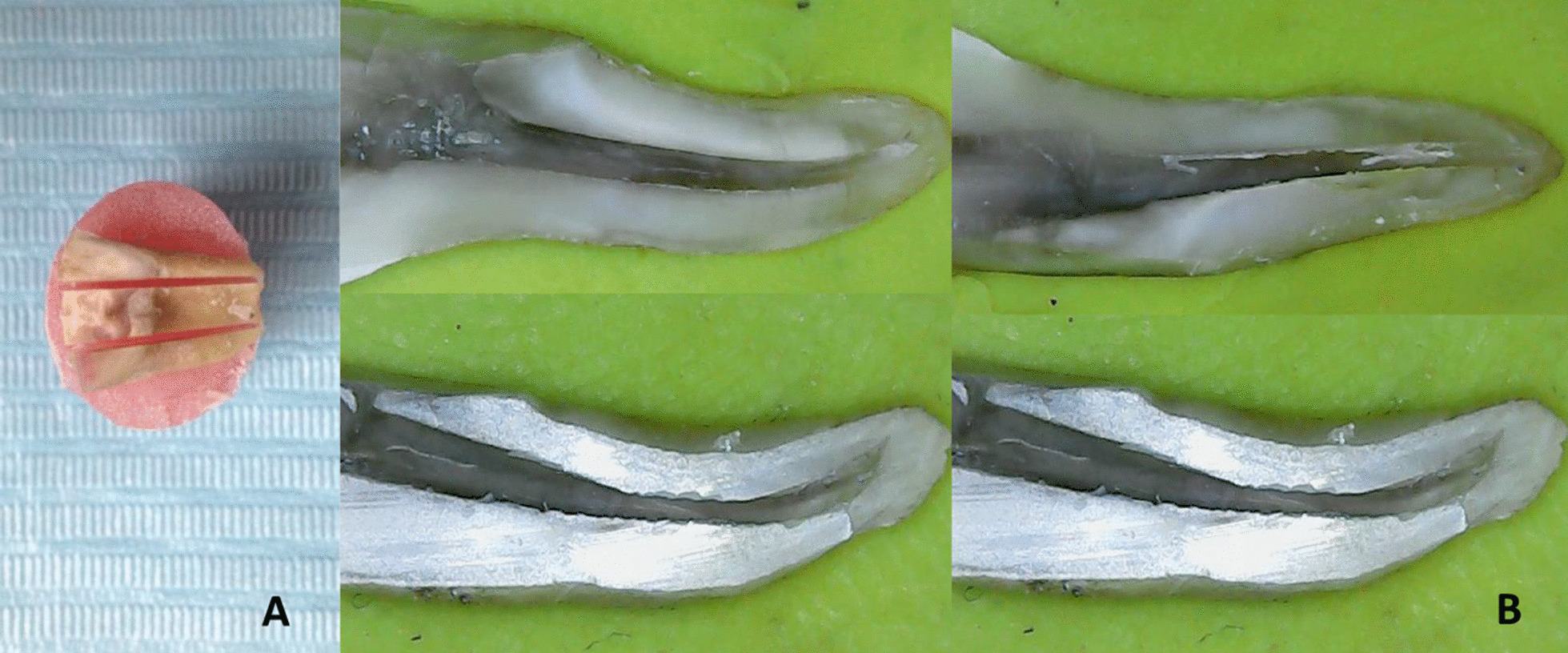
**A** showing sections of mesiobuccal and mesiolingual root canals in a mesiodistal direction. **B** stereomicroscopic microphotographs at ×30 showing sections of mesiobuccal and mesiolingual root canals

A new sharp razor blade was inserted. With a gentle tap on the root and two fingers secured, the root was split into three longitudinal parts in the mesiodistal direction (Fig. 1A).

After retreatment, the percentage of residual filling material in the coronal, middle, and apical segments was evaluated using a stereomicroscope (Scope Capture, China) and ImageJ software (National Institutes of Health, v1.39a).

Images of each segment were captured using a USB stereomicroscope connected to an IBM computer at 30X magnification to allow clear visualization of the canals. The total area of each segment was calculated, and the percentage of the area covered by the obturating material (gutta-percha/sealer) remnants was calculated using ImageJ software (Fig. 1B) [20].

The residual filling area of the segment is detected, outlined, and measured. The total canal area and the corresponding residual filling material areas were determined. The coded images were identified and outlined using specific software tools with the agreement of three trained examiners who were blinded to the group assignment. The percentage of the residual filling material was calculated by dividing the filling material-covered areas by the designated canal areas and multiplying by 100.

### Statistical analysis

The Shapiro–Wilk test was used to ensure that the data were normally distributed. The data were nonparametric and did not follow a normal distribution. Mean, standard deviation, median, and minimum and maximum values were all descriptive statistics. To compare the groups, the Mann–Whitney U test was used. To compare the percentages of residual filling material between the teeth segments, the Kruskal–Wallis test was used, followed by the Mann–Whitney test for multiple comparisons between the two segments (post hoc test). The statistical significance level was set at p < 0.05. The data were analyzed using Statistical Package for Social Science, version 25.

## Results

A comparison of the residual filling material percentages between the groups and the tooth segments is presented in Table 1. For the coronal and middle segments, there was a significant difference in the median percentage of residual filling material between the groups (P < 0.001), but for the apical segment, there was no significant difference in the median percentage of residual filling material between the groups. For the coronal and middle segments, the R-Endo group showed a significantly higher median percentage of residual filling material than did the XPS group.

**Fig. 2:**
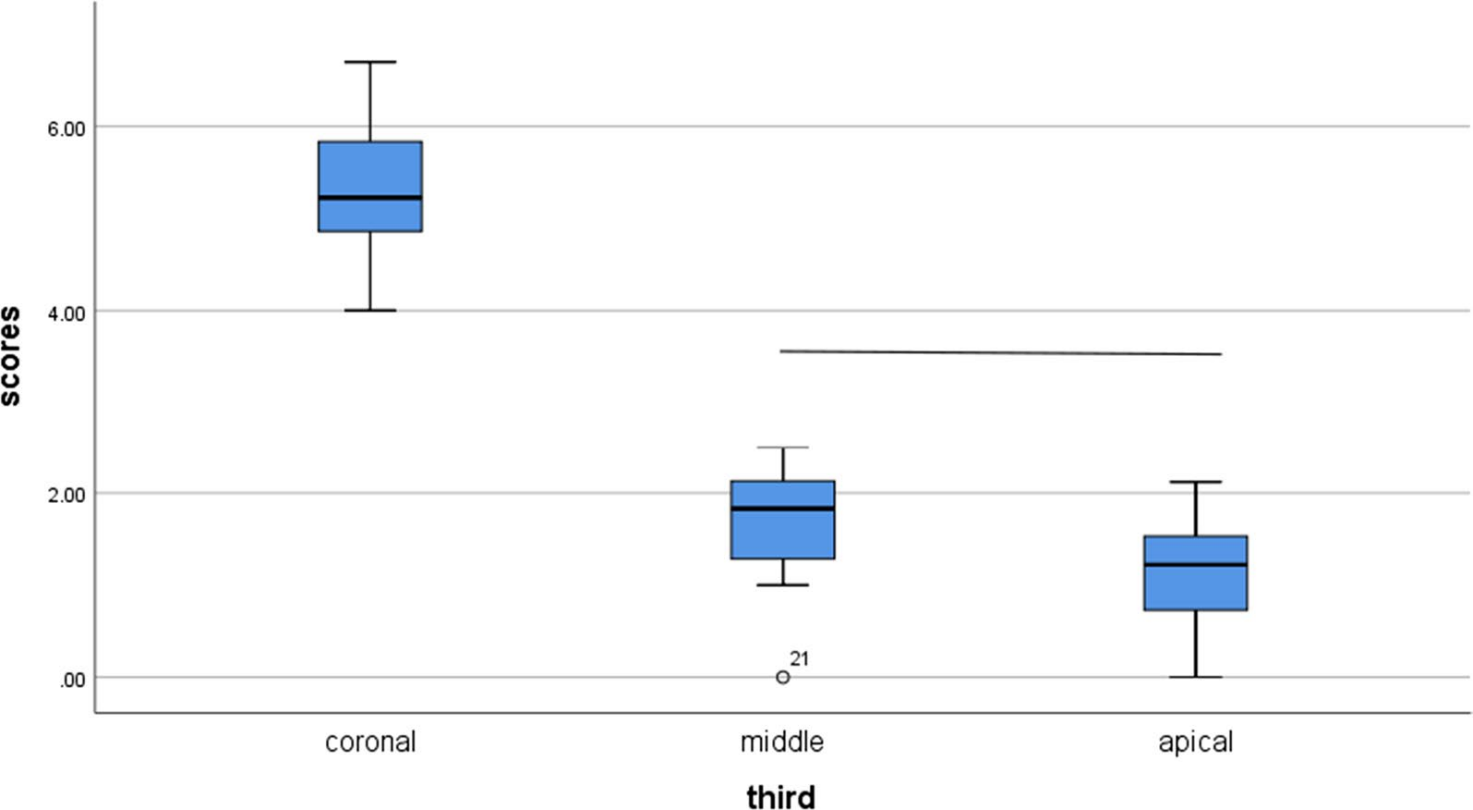
Multiple comparison of median percentages of residual filling material between different segments of the teeth for the XP-endo Shaper group. Line connecting boxplots indicate no significant differences between each 2 segments of the teeth

**Fig. 3:**
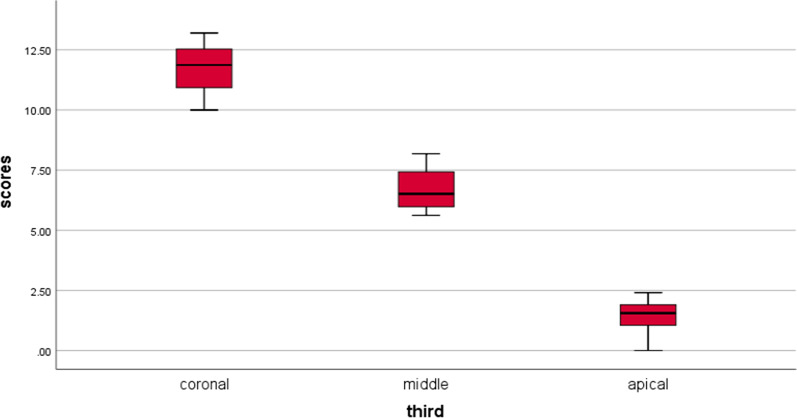
Multiple comparison of median percentages of residual filling material between different segments of the teeth for the R-Endo group. Line connecting boxplots indicate no significant differences between each 2 segments of the teeth

The median percentage of residual filling material differed significantly between tooth segments in both the XPS and R-Endo groups (p < 0.001, Table 1). Multiple comparisons of the medians of the residual filling material percentages between the different segments of the teeth in both groups are presented in Table (1) for the XPS group shown in Fig. 2 and for the R-Endo group shown in Fig. 3. For both groups, the coronal segment had the highest median for the residual filling material, followed by the middle segment; the apical segment showed the lowest median. In the XPS group, a significantly higher median amount of residual filling material was detected in the coronal segments. However, no significant difference in the median value was found between the middle and apical segments. In the R-Endo group, there was a significantly higher median between each of the two tooth segments.

**Table 1:** Comparison of residual filling material percentages between groups and teeth segments

	XP-Endo					R-Endo					Mann–Whitney test (P value)
	*X*	*SD*	*M*	*min*	*max*	*X*	*SD*	*M*	*min*	*max*	
Coronal	5.28a	0.73	5.23a	4.00	6.70	11.77a	0.92	11.87a	10.00	13.20	< 0.001*
Middle	1.70b	0.61	1.83b	0.00	2.50	6.70b	0.87	6.52b	5.62	8.18	< 0.001*
Apical	1.16b	00.60	1.22b	0.00	2.12	1.44c	0.61	1.56c	.00	2.41	0.189
Kruskal–Wallis test (P value)	< 0.001*					< 0.001					

*X* mean, *SD* standard deviation, *M* median, *min* minimum, *max* maximum

*P is significant at the 5% level of significance. Different letters in the same column show significant differences between each 2 segments of the teeth (Mann–Whitney test, P < .05). The same letters in the same column show nonsignificant differences between each 2 segments (Mann–Whitney test, P > .05)

## Discussion

The persistence of microbial infections in root canals is considered a major factor in endodontic failure, and nonsurgical retreatment is the initial treatment of choice [3]. The success rate of retreatment may be related to many factors, including tooth type, canal anatomical complexity, and the techniques used for filling material removal. Retreatment procedures include the removal of the residual infected filling materials that induce failure to enable chemomechanical instrumentation and root canal disinfection [21].

The objective of this study was to evaluate the retreatment efficacy of XP-endo Shaper and R-Endo files in curved root canals using ImageJ software.

The mesial roots of Vertucci class IV mandibular molars were examined in this study. Mesial roots are particularly intriguing not only because of the anatomical complexity of the typically narrow and curved canals but also because of the opportunity to test the two systems in the same root. This approach reduced the bias associated with canal selection [22]. Removing the filling material and additional canal preparation are time-consuming and more complex in curved canals than in straight ones. When accentuated, these curvatures may lead to complications during clinical procedures, compromising the safety and integrity of the instruments used for treatment. A small angle and radius of root canal curvature are considered to be important factors that increase the tensional load on rotary nickel-titanium (NiTi) instruments and, consequently, the risk of operational accidents such as apical deviation, perforation, and instrument separation [23].

Various methods have been used to assess the amount of root canal filling material found inside the canals following retreatment. Radiography was used to estimate the residual root-filling material. To evaluate filling material remaining in root canals, radiography is commonly used. However, radiographic examination yields a two-dimensional representation of a three-dimensional structure that can be magnified and distorted. To avoid the shortcomings mentioned above, a stereomicroscopic imaging technique at 30X magnification was used to obtain a clear image of the residual obturating material, as recommended by Schirrmeister et al. [21]. The surface area of the root canal segments was calculated using ImageJ software, and the percentage of residual filling material was determined. This method is efficient because it is simple to use and maintains a constant distance between the device and the object, allowing image standardization [24].

The dentin bond strength has been shown to be higher in bioceramic sealers than in resin-based sealers. The Sure-Seal Root used in this study is a bioceramic sealer with a high dentinal bond strength, which makes removal from the dentinal walls during the retreatment procedure more difficult [25].

R-Endo retreatment rotary system was chosen for this study on the basis of efficacy in removing gutta-percha from the root canal during endodontic retreatment [26]. R-Endo was compared to XP Endo Shaper which has a snake-like shaped file to maximize the efficacy of cleaning the root canal system with the ability to touch most of the canal's walls. This system can reportedly be used in initial treatment and retreatment [27].

The results of this study showed that XPS files were significantly more effective than R-Endo files in removing root canal fillings from coronal, and middle segments. In both groups, the coronal segments had the highest percentage of residual filling material, followed by the middle and apical segments. The use of at least one diameters greater than the last instrument previously used, seems to represent an adequate balance between improving removing of obturating materials at the apical segment and conservation of tooth structure. Nevertheless, during root canal retreatment, excessive removal of dentin should be avoided, in order to decrease the risk of perforations, cracks and vertical fractures [28]

Endodontic retreatment can benefit from XPS. The manufacturer recommends running the XPS at 800–1000 rpm with 1 Ncm torque. They cannot, however, penetrate or progress through the filling material when used in the recommended settings. According to several pilot studies [17], increasing the speed up to 3000 rpm enhanced penetration ability and retreatment efficiency while causing no additional unfavorable events.

XPS had a slender profile with a narrow taper and a booster tip that expanded at body temperature. The improved XPS efficiency can be credited to the file design and high-speed plasticization of gutta-percha, which may facilitate its removal [29].

Garg et al. [18] advocated R-Endo retreatment files with active cutting tips, which aid in the initial progression through the gutta-percha and provide adequate cleaning while respecting the canal anatomy.

The residual filling material found in the canal is reduced when canal enlargement during the retreatment procedure is greater than that performed prior to root canal obturation [30]. As a result, the retreatment procedure was carried out using an instrument one size larger for XPS (30/0.04) than the enlarged size used during primary preparation X2 (25/0.06). On the other hand, R-Endo retreatment ended at R3 (25/0.04).

The findings of this study support the findings of De-Deus et al. [6], Emre et al. [31], and Kapasi et al. [32] that improved XPS files are more effective than other systems in removing filling materials.

## Conclusion

It can be concluded that, under the limitations of this study, all retreatment techniques left some filling material inside the root canal. XP-endo Shaper is significantly more effective than R-Endo in removing root canal filling materials in the coronal, middle, and apical segments. Further studies with larger sample sizes are essential to determine the retreatment efficiency regarding the time of removal and the amount of residual filling materials compared to other methods of evaluation, such as micro-CT and scanning electron microscope.

## Data Availability

The datasets generated and analyzed during the current study are not publicly available due to (ownership of data) but are available from the corresponding author on reasonable request.
